# Evaluating Large Language Models for Automated Evidence Synthesis in Neuroimaging AI: A Multi-Model Benchmark

**DOI:** 10.3390/jcm15114230

**Published:** 2026-05-30

**Authors:** Umid Sulaimanov, Nafiye Sanlier, Ariorad Moniri, Behman Demir, Yerkebulan Serikkanov, Ahmed Rasim Bayramoglu, Maryam Sabah Al-Jebur, Melih Yucel Sanlier, Ugur Erginoglu, Erkin Otles, Simon Gashaw Ammanuel, Abdullah Keles, Ufuk Erginoglu, Mustafa Kemal Baskaya

**Affiliations:** 1 Department of Neurological Surgery, School of Medicine and Public Health, University of Wisconsin-Madison, Madison, WI 53792, USA; 2 School of Medicine, Acibadem University, Istanbul 34752, Turkey; 3 Department of Emergency Medicine, Istanbul Medeniyet University, Prof. Dr. Suleyman Yalcin City Hospital, Istanbul 34722, Turkey; 4 Department of Otaryngology-Head and Neck Surgery, Basaksehir State Hospital, Istanbul 34480, Turkey; 5 BerbeeWalsh Department of Emergency Medicine, School of Medicine and Public Health, University of Wisconsin-Madison, Madison, WI 53792, USA

**Keywords:** artificial intelligence, benchmarking, evidence synthesis, information extraction, large language models, neuroimaging

## Abstract

**Background**: Data extraction for systematic reviews is highly resource-intensive. This study evaluated four frontier large language models (LLMs) on complex structured metadata extraction from specialized neuroimaging artificial intelligence (AI) literature to determine their performance in automated evidence synthesis. **Methods**: We compared Google Gemini 3 Pro Preview, Anthropic Claude Opus 4.5, Perplexity Sonar Pro, and OpenAI GPT 5.2. Using a standardized prompt, each model extracted 22 variables from 91 peer-reviewed neuroimaging AI articles. The variables were stratified into low-, medium-, and high-complexity tiers. The performance was measured via the exact-match accuracy against a consensus-based expert ground truth. **Results**: The overall exact-match accuracy was moderate. Gemini 3 Pro Preview achieved the highest overall rate (56.4%), followed by Sonar Pro (52.1%), Claude Opus 4.5 (51.3%), and GPT 5.2 (46.5%). Gemini significantly outperformed all other models (*p* < 0.001). The performance declined dramatically as the variable complexity increased. Across models, the accuracy was 88.9–92.9% for low-complexity categorical fields, 47.0–63.3% for medium-complexity text extraction, and 2.7–15.5% for high-complexity variables requiring clinical judgment or multi-section synthesis. The most common type of error was misclassification. All four models scored 0% on the main performance metric, but this reflected a representational mismatch with the ground truth rather than extraction failure, indicating that the exact-match accuracy underestimates the true semantic performance. **Conclusions**: Frontier LLMs can effectively automate the retrieval of simple categorical data, but have serious difficulties with methodological variables that are complex. Although extraction can be fully automated for low-complexity fields, human review remains essential for context-dependent variables that require clinical judgment.

## 1. Introduction

The volume of medical literature is now growing faster than researchers can reliably synthesize manually. Systematic reviews, the gold standard for synthesizing evidence, require researchers to manually sift through hundreds of studies, extract structured data, and do it consistently across reviewers. Of all the steps in this process, data extraction stands out as particularly burdensome. It requires up to 125 min per study with human-only approaches [1]. Errors still find their way into up to 67% of meta-analyses [2]. The problem is not a lack of effort; it is that the task is inherently tedious, detail-intensive, and difficult to scale.

Large language models offer a promising way forward. Earlier natural language processing approaches required large amounts of labeled training data and struggled to generalize across tasks [3]. In contrast, modern LLMs can be prompted to extract structured information from unstructured scientific text without any task-specific training. The early results have been encouraging: AI-assisted extraction has been shown to match or exceed human accuracy while reducing the extraction time by a median of 41 min per study [1]. In more narrowly defined settings, the accuracies have been even higher. GPT-4 achieved above 97% for structured radiographic variable extraction from radiology reports [4,5,6]. Beyond data extraction, LLMs have been shown to facilitate screening reviews of papers for systematic reviews and have the potential to democratize other aspects of medical research. For example, they enable junior clinicians with no prior programming experience to complete AI projects at rates nearly three times higher than those achieved without LLM assistance [7].

Despite this progress, several key limitations exist in the available evaluations. Most have relied on earlier model generations or focused on narrow extraction tasks with limited variable sets and small domain-specific datasets [8,9]. How current frontier models perform on complex, multi-variable extraction tasks remains unclear. Such tasks require not only information retrieval, but also synthesis across multiple document sections and domain-level judgment.

Assessing the AI neuroimaging literature presents exactly this kind of challenge. Articles in this domain report on diverse architectures, heterogeneous validation approaches, and multiple dimensions of methodological quality, including the data leakage risk, split description clarity, and CLAIM/TRIPOD+AI adherence. These elements cannot be extracted by locating a single sentence. Standardized reporting frameworks, including the Checklist for AI in Medical Imaging (CLAIM) and TRIPOD+AI guidelines, define what should ideally be reported. However, adherence remains inconsistent, and extracting even well-defined variables from these articles demands a degree of judgment that may challenge current models [10,11].

A recent systematic methodological review audited 91 AI neuroimaging studies from 2025 for methodological quality [12]. The present study leverages that corpus to evaluate whether frontier LLMs can replicate such a structured extraction. We compared the performance of four frontier LLMs—Google Gemini 3 Pro Preview, Anthropic Claude Opus 4.5, Perplexity Sonar Pro, and OpenAI GPT 5.2—on the extraction of 22 structured metadata variables from 91 peer-reviewed AI neuroimaging articles published in 2025. We evaluated the overall and variable-level accuracy against an expert-derived ground truth, stratified the performance by variable complexity, and analyzed model-specific error profiles. Our goal was to characterize the performance of frontier LLM-based structured extraction in neuroimaging AI literature, and to identify where LLM-based extraction is ready for integration into research protocols and where human expertise remains essential.

## 2. Materials and Methods

### 2.1. Study Design and Dataset

We conducted a systematic inter-rater reliability analysis evaluating the ability of four large language models to extract structured metadata from AI neuroimaging research articles. The 91 peer-reviewed neuroimaging AI articles analyzed in this benchmark were drawn from the corpus of our previously published systematic methodological review, which followed the PRISMA guidelines and was prospectively registered with PROSPERO (CRD420261284068) [12]. The full search strategy, eligibility criteria, and PRISMA flow diagram are reported there. All articles addressed machine learning, deep learning, or hybrid applications in clinical neuroimaging with sufficient methodological detail for CLAIM/TRIPOD+AI-based extraction.

### 2.2. AI Models and Application Programming Interface Implementation

Four frontier large language models were evaluated: Google Gemini 3 Pro Preview (google/gemini-3-pro-preview), Anthropic Claude Opus 4.5 (anthropic/claude-opus-4.5), Perplexity Sonar Pro (perplexity/sonar-pro), and OpenAI GPT 5.2 (openai/gpt-5.2). All the models were accessed through OpenRouter, which provides standardized access to multiple LLM providers through a unified interface. This ensured consistent parameters and eliminated platform-specific implementation differences that could confound comparisons.

All 91 PDFs were preprocessed through MistralOCR to ensure identical plain-text input across models, eliminating bias from differential native PDF-parsing across LLM APIs. All the included articles were born-digital, publisher-issued PDFs containing an embedded text layer; no scanned or image-only documents were included. The OCR output was validated through automated checks (total character count, presence of expected section headings, and detection of typical OCR artifacts such as long non-ASCII runs or repeated character sequences) and a manual spot-check of articles by two reviewers, with particular attention to the methods, performance tables, and results sections. No systematic OCR failures were observed.

All four models were queried via the OpenRouter chat-completion API using identical sampling parameters: temperature = 0.3, top-p = 1.0, top-k = 0 (disabled), frequency penalty = 0.0, presence penalty = 0.0, repetition penalty = 1.0, min-p = 0.0, top-a = 0.0, and max output tokens = 0 (unrestricted). Model-specific reasoning and extended-thinking modes were not enabled, and for all models, no tool use, browsing, or function calling was permitted. Each article was submitted as a single-turn request, with the OCR-derived plain text submitted as a single user message; no truncation or chunking was applied, and all articles fit within every model’s context window. The models received OCR-derived plain text only; original images, figures, and table layouts were not provided as separate inputs. A retry policy allowed up to three attempts with exponential backoff (2 s initial delay, 2× multiplier). The exact model snapshot identifiers and API access dates were: OpenAI GPT 5.2 (openai/gpt-5.2-2025-12-11), Anthropic Claude Opus 4.5 (anthropic/claude-opus-4-5-20251101), Google Gemini 3 Pro Preview (google/gemini-3-pro-preview), and Perplexity Sonar Pro (perplexity/sonar-pro); all queries were executed on 2 February 2026. The full prompt and normalization rules are provided in Supplementary Data S1.

### 2.3. Ground Truth Development

The reference standard was derived from the consensus extraction dataset previously developed for the same 91-article corpus by three independent reviewers using a CLAIM/TRIPOD+AI-aligned form [12]. Because exact-match evaluations are sensitive to minor inconsistencies in the reference, the original ground truth underwent an additional curation pass: two reviewers independently re-examined all 22 variables across all 91 articles against the source PDFs, with a third reviewer adjudicating residual disagreements. This refined dataset served as the reference standard for all the analyses reported here.

### 2.4. Data Extraction

Using standardized AI reporting guidelines and methodological quality assessment frameworks, a standardized extraction schema made up of 22 variables was created. The schema consisted of eight study characteristics such as the first author’s name (surname and initials), publication year, journal, country where the dataset was produced, medical field, imaging modality, dataset type (single-center, multi-center, or public), and sample size. There were 14 methodological variables, namely the definition of ground truth, type of AI (deep learning, machine learning, or hybrid), model architecture, type of task (classification, detection, segmentation, prediction, diagnosis, identification, synthesis, grading, or multi-task combinations), method of validation (internal, external), use of external dataset (yes/no), clarity of split description (yes/no), risk of data leakage (low, moderate, high, or unclear), study design (retrospective, prospective, or mixed), presence of human comparator (yes/no), calibration metrics report (yes/no), definition of performance metrics (yes/no), adherence to CLAIM/TRIPOD+AI (yes, partial, no, or not reported), report of main performance metric, and claim of clinical applicability (yes/no). Variables were stratified into three complexity tiers based on the nature of the extraction task. Low-complexity variables (n = 7) involved fixed categorical variables that need the least interpretation: the year, AI type, claim of clinical applicability, performance metrics as defined, study design, external dataset used, and calibration metrics. Variables with a medium complexity (n = 8) necessitated text extraction and entity recognition with little interpretation: the human comparator, dataset type, country of dataset origin, risk of data leakage, type of task, imaging modality, medical field and journal. Variables with a high complexity (n = 7) required extraction in more than one document section, clinical inference or domain-level judgment. These variables were the sample size, CLAIM/TRIPOD+AI adherence, split description clarity, ground-truth definition, model architecture, type of validation, and primary reported performance metric.

### 2.5. LLM Query Pipeline

All of the article PDFs were programmatically posted to each of the four models through OpenRouter with the same structured JavaScript Object Notation (JSON) prompt. The prompt was used in a zero-shot setup without few-shot instances or explicit instructions to give a chain of thought. The prompt defined the desired form of output as a single-row markdown table. The table had the appropriate number of columns (22) in a predetermined sequence corresponding to the extraction schema. Fine-grained extraction guidelines advised the models to only extract information that is explicitly mentioned in the PDF and not assume anything not mentioned. Any missing information would be marked with the standardized expression of not reported. The models were also advised to have concise answers and they should not speculate or assume. Each variable had detailed field definitions in its prompt. The specified definitions permitted categorical values, exemplary cases, and clear decision criteria in case of ambiguous cases. The prompt emphasized strict adherence to evidence-based extraction. This method is similar to the principle-based technique applied to ground truth development by reviewers. The complete prompt as submitted to all four models, including the system role specification, task definition, output requirements, full field definitions, allowed categorical values for each of the 22 variables, and normalization rules, is provided verbatim in Supplementary Data S1.

**Figure 1:**
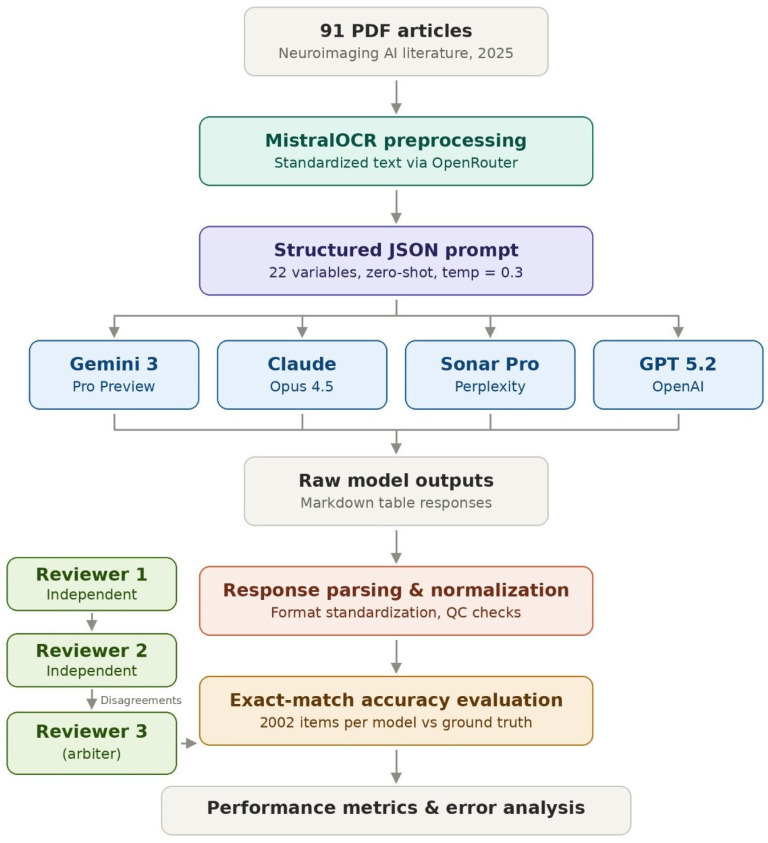
**Structured metadata extraction pipeline.** Ninety-one neuroimaging AI articles were preprocessed via MistralOCR, then submitted to four frontier LLMs using an identical structured JSON prompt (22 variables, zero-shot, temperature = 0.3). The model outputs were parsed, normalized, and evaluated against an expert consensus ground truth previously established by three independent reviewers and subsequently curated for an exact-match evaluation by two reviewers, with a third resolving residual disagreements.

The calls were logged with timestamps and model version identifiers. Full parameter specifications were also kept so that they could be reproducible. The complete extraction pipeline is illustrated in Figure 1. Failed queries were identified when errors occurred, including timeouts, API unavailability, or rate limitations. These queries were automatically retried 3 times with exponential backoff. Retries with persistent failures were put on manual review. These cases were subsequently resubmitted.

### 2.6. Response Processing and Quality Control

The model responses were systematically checked by validation to ensure integrity of the data. Each reply was parsed to retrieve the markdown table row. Automated tests ensured that the responses contained well-formatted tables having the right columns. A secondary parsing attempt was made on responses that did not pass an initial parsing attempt. There were alternative parsing rules to accommodate some small differences in format. The responses that were successfully parsed were normalized to ground-truth-encoding schemes. Categorical variables were standardized to fixed value sets, and continuous variables were verified to be in the expected format. Known abbreviations and spelling variants were normalized to canonical forms (e.g., “DL” to “deep learning”). Numeric values were compared after removing formatting characters (e.g., “n = 1234” and “1234” were treated as equivalent). Quality assurance and error analyses were supported by logging all processing steps. This included parsing failures and normalization transformations.

### 2.7. Statistical Analysis

The primary outcome measure was the exact-match accuracy. For each model, this was calculated as the proportion of extracted items that matched the ground truth reference: (number of items matching GT) ÷ (total items evaluated). Items were excluded only when a model’s response could not be parsed (0–1 items per model). This resulted in an evaluation of N = 2002 (91 articles × 22 variables) items per model. All proportions were calculated using Wilson score 95% confidence intervals. No continuity correction was done. Cohen’s κ was used to assess each model’s agreement with the ground truth and to measure the overall system agreement. This provided four model-to-GT comparisons. Each of the κ values was read as per the benchmarks of Landis and Koch. These categories were defined as follows: <0.00 (poor), 0.00–0.20 (slight), 0.21–0.40 (fair), 0.41–0.60 (moderate), 0.61–0.80 (substantial), and 0.81–1.00 (almost perfect agreement).

There were three mutually exclusive error categories. These were exact matches (model response matched ground truth), omissions (model returned “not reported” when ground truth had a valid value), and misclassifications (model returned an incorrect value that was neither a correct match nor an omission). The rates of omission and misclassification were determined as proportions of erroneous items only. In this study, hallucination was not evaluated.

Stratified analyses of the performance differences were conducted in various dimensions. A variable complexity analysis involved chi-square tests to determine any significant differences in the accuracy between the low-, medium-, and high-complexity tiers at each model. The accuracy distributions per article were examined to determine systematic variation in the model performance across the 91-article corpus, and descriptive statistics (mean, median, SD, range) were computed on article-level exact-match rates in each model.

Because the four models were evaluated on the same articles and variables, the data followed a repeated-measures design. The omnibus comparison of the overall exact-match accuracy therefore used Cochran’s Q, the appropriate test for dependent binary outcomes. The Friedman test, the non-parametric repeated-measures analog of a one-way ANOVA, was used to compare the distributions of the article-level accuracy. Wilcoxon signed-rank tests with a Bonferroni correction (6 pairs, α-threshold = 0.0083) were performed as post hoc pairwise tests. In each level of complexity, Cochran’s Q tests were used to compare the differences in accuracy between the models. Statistical significance was defined as *p* < 0.05 for all analyses.

To further address the nested structure of the data (2002 items per model are grouped by article and by variable rather than being independent), a sensitivity analysis was conducted using Bayesian variational-Bayes logistic mixed-effects regression, with the AI model and variable complexity tier as fixed effects and crossed random intercepts for article (n = 91) and variable (n = 22). Odds ratios with 95% confidence intervals were computed relative to the lowest-performing model (GPT 5.2) and the lowest-complexity tier as reference categories.

## 3. Results

### 3.1. Overall Model Performance

**Figure 2:**
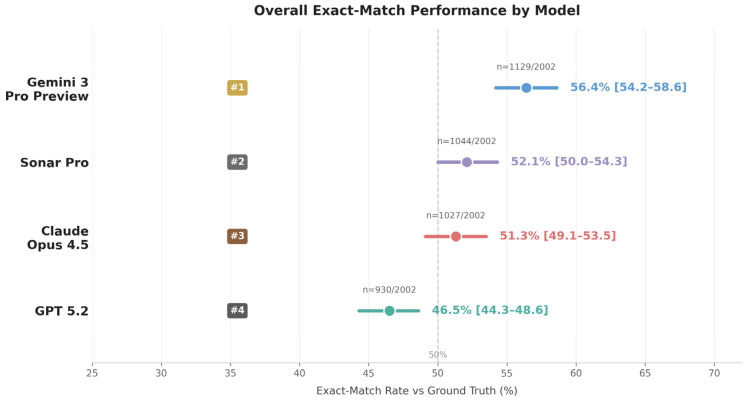
**Overall exact-match performance by model.** Dot plot showing exact-match accuracy (%) against expert ground truth for four frontier LLMs across 91 articles and 22 variables (N = 2002 items per model). Horizontal bars represent 95% Wilson confidence intervals; dashed line indicates 50% threshold. Models are ranked by performance. Cochran’s Q = 143.58, df = 3, *p* < 0.0001. Claude Opus 4.5 and Sonar Pro were statistically indistinguishable (*p* = 1.000); all other pairwise comparisons were *p* < 0.001.

The four AI models were assessed on 91 articles, resulting in 2002 items per model (91 articles × 22 variables; 0–1 items were excluded because of unparseable responses). The exact-match accuracy differed significantly across models (Cochran’s Q = 143.58, df = 3, *p* < 0.0001). Gemini 3 Pro Preview had the best total exact-match rate at 56.4% (95% CI: 54.2–58.6%). Sonar Pro followed at 52.1% (50.0–54.3%), followed by Claude Opus 4.5 at 51.3% (49.1–53.5%). GPT 5.2 showed the lowest performance at 46.5% (44.3–48.6%). These results are summarized in Table 1 and Figure 2. Wilcoxon signed-rank tests were used to perform follow-up pairwise comparisons with a Bonferroni correction due to multiple comparisons. Gemini 3 Pro Preview showed a significant improvement over the other three models (all *p* < 0.001). There was no statistical difference between Claude Opus 4.5 and Sonar Pro (*p* = 1.000). Compared to all other models (*p* < 0.001), GPT 5.2 performed significantly worse. None of the four models exceeded a 57% exact-match accuracy, indicating that a substantial proportion of structured metadata was extracted incorrectly or not at all, regardless of the model used.

To verify that the model ranking observed under the omnibus tests was not an artifact of treating clustered items as independent, we re-analyzed the data using mixed-effects logistic regression with crossed random intercepts for article and variable (Supplementary Table S2). The ranking and significance pattern were preserved: all three frontier models had significantly higher odds of correct extraction than GPT 5.2 (Claude Opus 4.5: OR 1.45, 95% CI 1.29–1.64; Sonar Pro: OR 1.55, 1.37–1.75; Gemini 3 Pro Preview: OR 2.17, 1.92–2.45; all *p* < 0.0001). Variable complexity emerged as a dominant predictor (medium vs. low: OR 0.11, 0.10–0.12; high vs. low: OR 0.005, 0.004–0.006), and the variance attributable to variables (1.20 log-odds^2^) substantially exceeded that attributable to articles (0.16).

### 3.2. Variable-Level Performance

**Figure 3:**
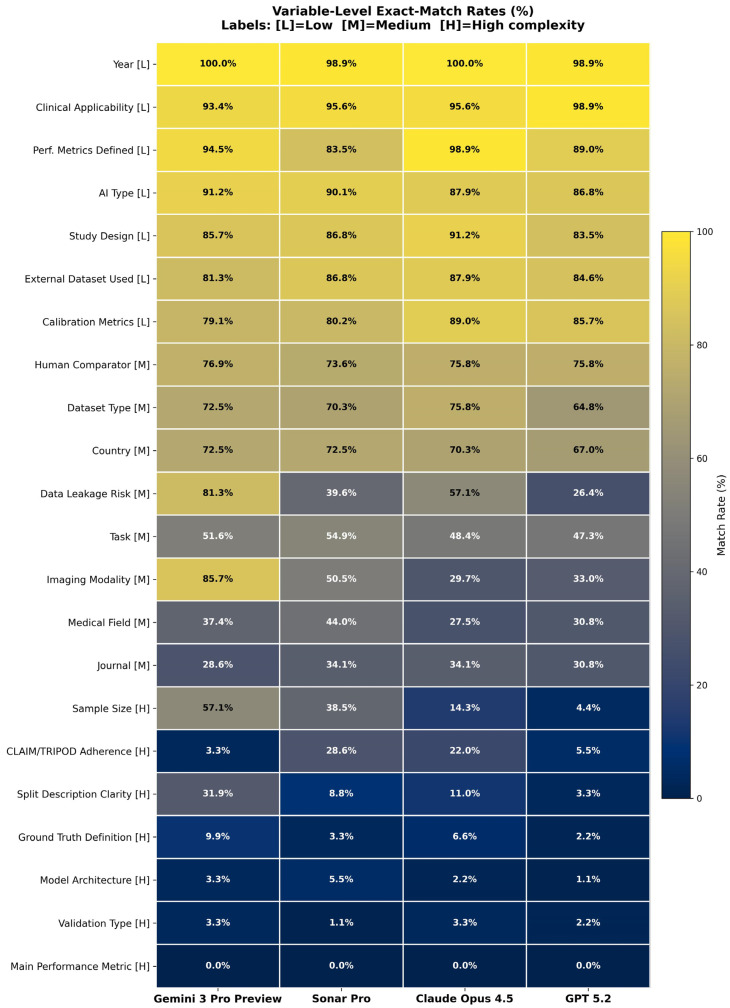
**Variable-level exact-match rates by model and complexity tier.** Heatmap of exact-match accuracy (%) for 22 extraction variables across four models. Variables are sorted by descending mean accuracy and labeled by complexity tier: low [L] (n = 7), medium [M] (n = 8), and high [H] (n = 7). Color ranges from dark blue (0%) to bright yellow (100%). Main performance metric [H] yielded 0% across all models under exact-match scoring due to representational differences in numeric format, reporting scope, and free-text descriptors; semantic content was correctly extracted by all models.

The variable-level performance, visualized as a heatmap in Figure 3, was sorted by descending mean accuracy and labeled by complexity tier. Three distinct performance bands emerged.

Low-complexity variables formed a high-performance cluster at the top of the heatmap. The year was extracted near-perfectly across all models (98.9–100%), as was the clinical applicability claim (93.4–98.9%). The performance metrics defined (83.5–98.9%), AI type (86.8–91.2%), and study design (83.5–91.2%) showed a similarly high accuracy. The external dataset used (81.3–87.9%) and calibration metrics (79.1–89.0%) completed the low-complexity tier with consistently high extraction rates across models. These variables share two properties—a small, well-defined value set and a single canonical location in the article—that, together, appear to enable reliable extraction.

Medium-complexity variables clustered in the middle band of the heatmap and showed pronounced inter-model heterogeneity. The human comparator (73.6–76.9%), dataset type (64.8–75.8%), and country of origin (67.0–72.5%) were extracted with a moderate accuracy across the models. In contrast, the data leakage risk showed the largest inter-model spread (26.4% for GPT 5.2 to 81.3% for Gemini 3 Pro Preview), and the imaging modality showed an analogous pattern (29.7% for Claude Opus 4.5 to 85.7% for Gemini 3 Pro Preview). The task type (47.3–54.9%), medical field (27.5–44.0%), and journal (28.6–34.1%) clustered at the lower end of the medium tier. Heterogeneity was visually striking in this band: cells for the same variable shifted from red to green depending on the model, indicating that medium-complexity extraction is highly model-dependent.

High-complexity variables formed a low-performance cluster at the bottom of the heatmap. The sample size showed the widest spread within this tier (4.4% for GPT 5.2 to 57.1% for Gemini 3 Pro Preview), reflecting a differential ability to disambiguate the primary cohort size from secondary numeric values. The CLAIM/TRIPOD+AI adherence (3.3–28.6%), split description clarity (3.3–31.9%), and ground-truth definition (2.2–9.9%) were extracted at low rates across all models. The model architecture (1.1–5.5%) and validation type (1.1–3.3%) were the most challenging variables. In contrast, the main performance metric yielded a 0% exact-match accuracy across all four models, reflecting representational differences (numeric format and reporting scope) between the model outputs and the ground truth rather than failure to identify the reported metric; under a semantic-equivalence criterion, all four models recovered the reported metric in 89.0–97.8% of articles (Supplementary Table S1).

Across the full heatmap, Gemini 3 Pro Preview occupied the green-shifted cells most consistently, while GPT 5.2 occupied the red-shifted cells most consistently. This visual pattern mirrors the overall model ranking observed in Section 3.1.

### 3.3. Performance by Variable Complexity

**Figure 4:**
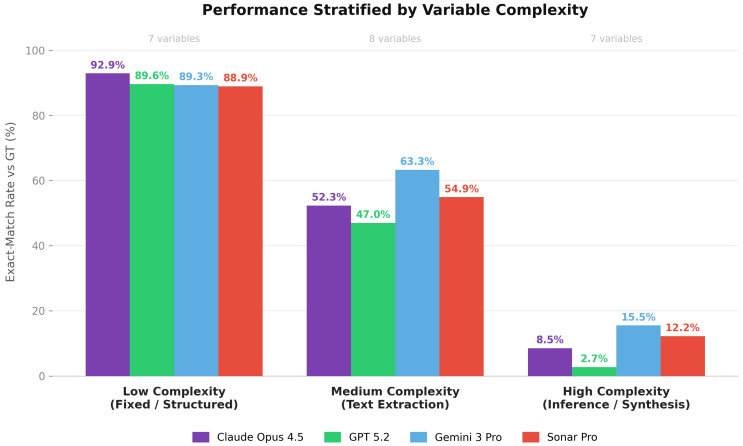
**Performance stratified by variable complexity**. Grouped bar chart of mean exact-match accuracy (%) by complexity tier: low (n = 7; fixed categorical), medium (n = 8; text extraction), and high (n = 7; inference/synthesis). Accuracy ranged from 88.9 to 92.9% (low), 47.0 to 63.3% (medium), and 2.7 to 15.5% (high). The between-model differences were significant at all tiers (Cochran’s Q: low, *p* = 0.002; medium and high, *p* < 0.0001).

Stratification by complexity tier revealed a consistent and dramatic performance gradient across all models (Table 2, Figure 4). For low-complexity variables (n = 7; fixed categorical items), the exact-match rates ranged from 88.9% to 92.9%, with Claude Opus 4.5 performing the highest (92.9%, 95% CI: 90.7–94.7%) and Sonar Pro the lowest (88.9%, 95% CI: 86.2–91.1%). The between-model differences at this tier were statistically significant (Cochran’s Q = 14.52, *p* = 0.002). However, these differences were modest in practical terms. For medium-complexity variables (n = 8), the performance declined substantially. These variables involved text extraction and entity recognition with minimal interpretation. The performance ranged from 47.0% (GPT 5.2) to 63.3% (Gemini 3 Pro Preview). The differences between the models were statistically significant (Cochran’s Q = 103.73, *p* < 0.0001). For high-complexity variables (n = 7), the performance was significantly worse. These variables needed multi-section collation, clinical inference, or cross-document judgment. The performance ranged from 2.7% (GPT 5.2) to 15.5% (Gemini 3 Pro Preview). There were statistically significant differences between models (Cochran’s Q = 95.23, *p* < 0.0001).

### 3.4. Error Taxonomy

Misclassification (incorrect value given) was the most prevalent across all models and was far more frequent than the other wrong extractions. It accounted for 97.3% of errors in Claude Opus 4.5, 90.6% in GPT 5.2, 89.5% in Gemini 3 Pro Preview, and 91.6% in Sonar Pro (Table 3). Omissions (model returned “not reported” when the ground truth contained a valid value) were uncommon with Claude Opus 4.5 (2.7% of errors), but more common with GPT 5.2 (9.4%), Gemini 3 Pro Preview (10.5%), and Sonar Pro (8.4%). Compared to GPT 5.2 (101) and Gemini 3 Pro Preview (92), Claude Opus 4.5 made just 26 omissions out of 2002 items, in absolute terms.

### 3.5. Inter-Rater Agreement

The full-vector Cohen’s κ values against the ground truth were within moderate agreement for all the models. Gemini 3 Pro Preview achieved the highest κ (0.502), followed by Sonar Pro (0.457), Claude Opus 4.5 (0.452), and GPT 5.2 (0.404). Some of the variables depicted close to zero κ, although the proportional accuracy was high, with the year and performance measures being the most prominent. This is an established statistical artifact that occurs when a response category is dominant (>98%). In this situation, apparent agreement is overblown whereas κ is underestimated. The κ that was estimated to be the lowest was the model architecture. This observation is in line with its low extraction rate in all the models.

### 3.6. Article-Level Accuracy Distribution

**Figure 5:**
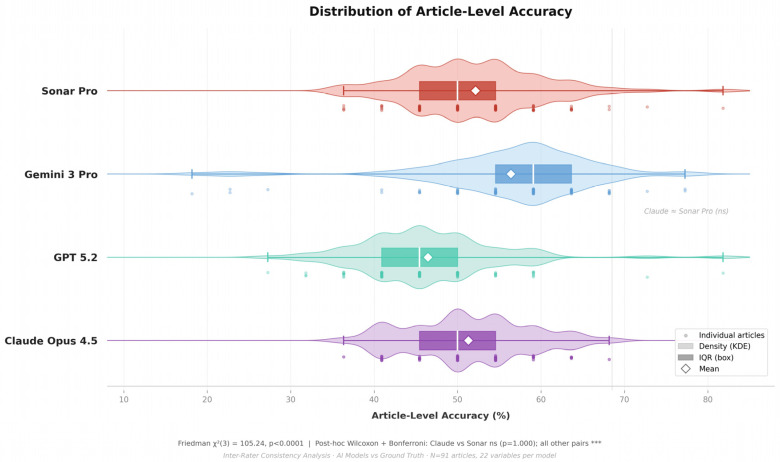
**Distribution of article-level accuracy.** Raincloud plots combining kernel density estimates, box plots, and individual data points for per-article exact-match accuracy across 91 articles. Diamond markers indicate the mean. No model exceeded 82% on any article. Friedman χ^2^(3) = 105.24, *p* < 0.0001. Claude Opus 4.5 and Sonar Pro were statistically indistinguishable (*p* = 1.000); for all other pairs, *p* < 0.001. *** denotes *p* < 0.001.

There was a significant difference in the article-level accuracy distributions between models (Friedman χ^2^(3) = 105.24, *p* < 0.0001) (Figure 5). Gemini 3 Pro Preview had the best mean article-level accuracy (56.4%), as well as median (59.1%). It also showed the widest distribution (SD = 10.1%, range: 18.2–77.3%). This is an indication of an increased variability among articles. Claude Opus 4.5 (mean: 51.3%, median: 50.0%, SD: 7.3%) and Sonar Pro (mean: 52.1%, median: 50.0%, SD: 8.1%) showed near-identical distributions. They statistically did not differ (Wilcoxon post hoc, *p* = 1.000 after Bonferroni correction). GPT 5.2 had the lowest mean article-level accuracy (46.5%, median: 45.5%, SD: 8.2%). None of the models had a higher accuracy than 82% on a single article. The lowest article-level accuracy of the models was 18.2% (Gemini 3 Pro Preview) to 36.4% (Claude Opus 4.5 and Sonar Pro). This means that even the most successful models performed significantly worse on some of the articles. Articles describing commercial black-box models, multi-task models with non-standard architectures, or studies with substantial gaps in reporting tended to yield a lower extraction accuracy across all models.

### 3.7. Domain-Level Accuracy Profile

**Figure 6:**
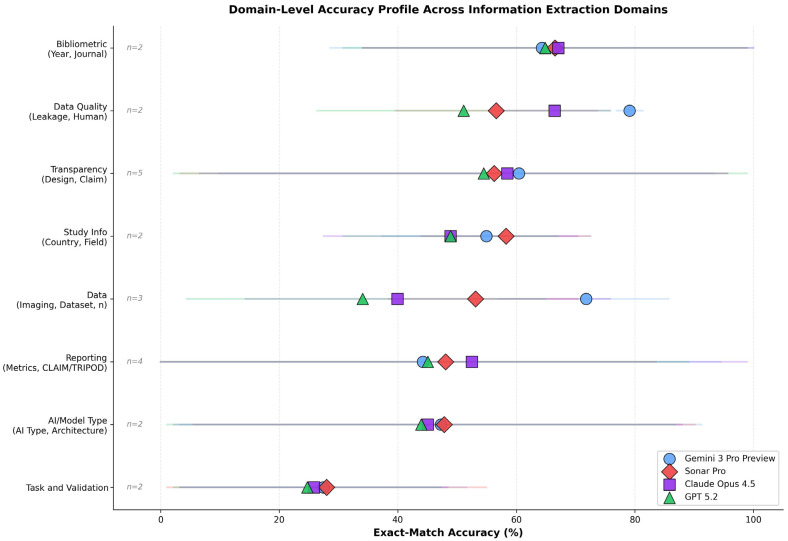
**Domain-level accuracy profile across information extraction domains**. Dot plot of the mean exact-match accuracy (%) across eight aggregated domains for the four large language models. The number of constituent variables (range: 2–5) is shown to the left of each row; the full domain composition is described in Section 3.7. Means are unweighted across constituent variables. Horizontal range bars indicate the minimum-to-maximum span within each domain for each model. Domains are sorted by the overall mean accuracy across models (highest at top).

A comparison of the eight aggregated information domains showed that all the models had a similar strength–weakness profile (Figure 6). The bibliometric domain (year, journal) and AI/model type domain showed the highest unweighted mean accuracies across the models. This reflects the relative ease of extracting well-defined categorical identifiers. Conversely, the reporting domain (performance metrics defined, CLAIM/TRIPOD+AI adherence, main performance metric, calibration metrics) and data quality domain (data leakage risk, human comparator, split description clarity) showed the lowest accuracies. This is consistent with the demands of inference-based extraction. Gemini 3 Pro Preview demonstrated particular strength in the data domain (imaging modality, dataset type, sample size). This was driven by its substantially higher extraction accuracy for imaging modality (85.7%) and sample size (57.1%) compared to other models. Overall domain-level rankings were consistent with the primary outcome results. Gemini 3 Pro Preview led across most domains, followed by Sonar Pro, Claude Opus 4.5, and GPT 5.2.

## 4. Discussion

Data extraction remains one of the most labor-intensive steps in evidence synthesis, requiring up to 125 min per study with human-only approaches [1]. It also contributes to errors in up to 67% of meta-analyses [2]. Given the rapid evolution of large language models, evaluating their current capabilities for this task remains essential. Prior evaluations have been limited to earlier model generations and smaller domain-specific samples [8,9]. This leaves a gap in understanding how current frontier models perform on complex, multi-variable extraction tasks in the specialized neuroimaging AI literature.

Our study addresses this gap by comparing four frontier models: Gemini 3 Pro Preview, Claude Opus 4.5, Perplexity Sonar Pro, and GPT 5.2. We compared their performance on structured metadata extraction from peer-reviewed neuroimaging AI articles. The overall exact-match accuracy ranged from 46.5% for GPT 5.2 to 56.4% for Gemini 3 Pro Preview. These values are substantially lower than those reported in prior LLM extraction studies. Gartlehner et al. found an accuracy exceeding 91% when extracting data in a systematic review context [1]. Choubey et al. reported GPT-4 accuracies above 97% for radiographic variable extraction from radiology reports [4]. These comparisons are, however, not straightforward. The models applied in previous research were mostly asked to find values in the form of clearly stated values in predictable document positions. Such variables as the CLAIM/TRIPOD+AI adherence, risk of data leakage, and clarity of split description have to be read in several parts. They also demand a high order of judgment as opposed to the mere retrieval of one precisely stated value. The performance gap observed here reflects the task complexity rather than inherent model limitations. It also highlights the fact that the performance of the extraction of the LLM cannot be assumed to generalize across domains without empirical validation.

### 4.1. Complexity-Stratified Performance and Its Implications

Perhaps the most practically relevant finding of this study is the steep decline in performance as variable complexity increased. On low-complexity categorical variables, the exact-match accuracy was between 88.9 and 92.9%. It decreased to 47.0–63.3% for the medium-complexity text extraction variables. The drop in performance was further, to 2.7–15.5%, for high-complexity variables that need multi-section synthesis, clinical inference, or the judgment of document sections. This gradient was the same in all four models and statistically significant at each tier. This implies that it is a mere property of the task and does not belong to a particular model. This is consistent with broader findings in the literature: LLMs perform well when prompted to retrieve well-defined values, but struggle when required to integrate contextual information or reconcile data reported inconsistently across sections [13,14,15,16].

The variables that reflect the most complex level entail the sample size, CLAIM/TRIPOD+AI adherence, clarity of split description, definition of ground truth, model architecture, type of validation, and main performance metric. These are also some of the most impactful variables in evidence synthesis in neuroimaging AI. The models did not perform well on these variables. This finding is of particular interest. This shows that completely automated extraction processes cannot be trusted with the variables that are of the greatest concern. Expert human review therefore remains essential in any pipeline that includes a methodological quality assessment.

### 4.2. Differential Model Performance and Characteristic Error Profiles

Gemini 3 Pro Preview achieved the highest overall exact-match accuracy (56.4%). It maintained superiority across all three complexity tiers. This was especially the case with medium- and high-complexity variables, such as the imaging modality (85.7%) and data leakage risk (81.3%). Nevertheless, the greatest article-level variability (SD = 10.1%, range: 18.2–77.3%) was also seen in Gemini 3 Pro Preview. This implies that there is more variability concerning its stronger average performance across articles. This ought to be taken into consideration when applying the model in practice. This is in line with the wider evidence on LLM output instability. In nominally deterministic conditions, the accuracy may differ up to 15% when repeated identical runs are performed. None of the models offer results that are reproducible in all tasks [17].

GPT 5.2 showed a consistently weaker performance across all complexity tiers. It had the lowest total exact-match rate (46.5%) and a near-zero accuracy on high-complexity variables (2.7%). In contrast, Claude Opus 4.5 and Sonar Pro did not exhibit any statistically significant differences between them in all of the pairwise comparisons (Wilcoxon *p* = 1.000). This implies that they provide capabilities for this kind of extraction task that are broadly similar. The most interesting difference between models was not in the overall accuracy. It emerged in how the models failed. Claude Opus 4.5 produced far fewer omissions than the other models, returning “not reported” only 26 times, compared to 80 for Sonar Pro, 92 for Gemini 3 Pro Preview, and 101 for GPT 5.2. In other words, Claude almost always returned a value, even when uncertain, whereas the other models more often returned “not reported.” This reflects two fundamentally different extraction strategies: Claude’s more assertive approach reduces the risk of missing information, but comes with higher chance of returning an incorrect value; omission-prone models are more conservative, but may systematically leave blank information that could have been filled. Neither strategy is inherently better; the right choice depends on whether the downstream workflow can more easily tolerate missing data or incorrect data. These patterns are broadly consistent with how frontier LLMs have been shown to differ in clinical and biomedical text-processing tasks more generally [14,18], and reflect the strategic trade-offs relevant to the design of human–AI extraction pipelines [19].

### 4.3. Representational Mismatches and the Apparent 0% Accuracy for the Main Performance Metric

A particularly notable finding was the apparent 0% exact-match accuracy for the primary performance metric variable across all four models. Despite this result, a qualitative review showed that the models consistently identified the correct metric and produced values that closely matched the ground truth. The discrepancies were largely representational, arising from differences in numeric formatting (such as decimal versus percentage notation), variations in the reporting scope (for example, a single primary value versus multiple values reported across sequences or cohorts), and the inclusion of free-text descriptions related to the validation context. Since exact-match scoring evaluates outputs through a strict character-by-character comparison, these semantically equivalent responses were consistently classified as mismatches. This finding underscores a key limitation of exact-match evaluations [20], which may significantly underestimate the true semantic extraction performance of free-text variables that can be expressed in multiple valid forms.

### 4.4. Limitations

Several limitations of this study should be noted. First, all four models were queried using a single structured prompt at a fixed temperature of 0.3, with no prompt optimization. More sophisticated approaches such as chain-of-thought reasoning, few-shot examples, or multi-turn refinement may yield a meaningfully better performance, particularly for high-complexity variables, but were beyond the scope of this study.

Second, the ground truth was constructed through a multi-reviewer consensus process and further curated for the present benchmark. The pre-consensus inter-rater agreement (e.g., Cohen’s κ), per-variable discrepancy proportions, and number of third-reviewer adjudications were not systematically logged, so the reference standard should be treated as a rigorous, but not perfect, benchmark.

Third, hallucinations, where a model generates a plausible, but factually unsupported, value, were not systematically assessed in this study. This is a meaningful gap, as hallucinated methodological quality data could have real consequences for evidence synthesis, and the extent to which they occurred here remains unknown.

Fourth, the corpus was limited to 91 AI neuroimaging articles, and the results may not generalize to other specialties or review types.

Fifth, although MistralOCR performed reliably on the born-digital PDFs used in this study, generalization of the pipeline to scanned or low-quality source documents would require a separate OCR robustness evaluation, which we did not undertake.

Sixth, the exact-match accuracy systematically underestimates the true semantic performance on free-text and numeric variables where multiple valid representations exist. This is most starkly illustrated by the 0% scores for the main performance metric, which reflected a representational mismatch rather than extraction failure. Accuracy figures should therefore be interpreted as conservative lower bounds, and semantic equivalence metrics (e.g., LLM as judge or embedding-based similarity) would be a useful complement in future work.

Finally, these findings reflect a snapshot of model capabilities as of 2 February 2026; given how rapidly the LLM landscape evolves, periodic re-evaluation will be essential.

## 5. Conclusions

This study demonstrates that current frontier LLMs achieve a moderate overall exact-match accuracy on structured metadata extraction from AI neuroimaging. The accuracy ranged from 46.5% for GPT 5.2 to 56.4% for Gemini 3 Pro Preview. The performance declined sharply as the variable complexity increased. Gemini 3 Pro Preview showed the best overall performance. GPT 5.2 was always found to be the worst in all analyses. Claude Opus 4.5 and Sonar Pro had very similar intermediate profiles.

The most informative was, perhaps, the 0% accuracy of the main performance metric of all models. This was not because of not comprehending the content. Instead, it was an encoding format mismatch between the ground truth and the model results that was systematic. This is a useful lesson that the benchmarks on the accuracy of the exact match can hide the real capabilities of the model. It also points out the fact that the prompt and schema design are as important as model selection.

When combined, these results argue in favor of a complexity-stratified method of LLM-assisted extraction. Low-complexity categorical variables seem to be reasonably automated. The pre-extraction that is aided by LLM and manually reviewed is more suitable to medium-complexity fields. Highly complex variables of methodology quality still depend on expert human extraction. Due to the ongoing development of LLMs, the line between reliably automatable tasks and those that must be supervised by a human is likely to change. Nonetheless, the facts of this study are clear: human expertise is still needed when it comes to making extraction decisions that are most important in building evidence.

## Figures and Tables

**Table 1:** **Overall exact-match performance by model**.

Model	Total Items (N)	Correct (n)	Rate (%)	95% CI (Wilson)
**Gemini 3 Pro Preview**	2002	1129	56.4%	54.2–58.6%
**Sonar Pro**	2002	1044	52.1%	50.0–54.3%
**Claude Opus 4.5**	2002	1027	51.3%	49.1–53.5%
**GPT 5.2**	2002	930	46.5%	44.3–48.6%

Note. N = 91 articles × 22 variables = 2002 evaluable items per model (0–1 items excluded per model due to unparseable responses). Rate = exact-match accuracy vs. expert ground truth. 95% CI computed using Wilson score method without continuity correction. Models ranked by overall performance. Cochran’s Q = 143.58, df = 3, *p* < 0.0001.

**Table 2:** **Performance by variable complexity level**.

Complexity	Model	N	Correct (n)	Rate (%)	95% CI (Wilson)
**Low (7 variables)**	**Claude Opus 4.5**	637	592	92.9%	90.7–94.7%
	**GPT 5.2**	637	571	89.6%	87.0–91.8%
	**Gemini 3 Pro Preview**	637	569	89.3%	86.7–91.5%
	**Sonar Pro**	637	566	88.9%	86.2–91.1%
**Medium (8 variables)**	**Gemini 3 Pro Preview**	728	461	63.3%	59.8–66.7%
	**Sonar Pro**	728	400	54.9%	51.3–58.5%
	**Claude Opus 4.5**	728	381	52.3%	48.7–55.9%
	**GPT 5.2**	728	342	47.0%	43.4–50.6%
**High (7 variables)**	**Gemini 3 Pro Preview**	637	99	15.5%	12.9–18.6%
	**Sonar Pro**	637	78	12.2%	9.9–15.0%
	**Claude Opus 4.5**	637	54	8.5%	6.6–10.9%
	**GPT 5.2**	637	17	2.7%	1.7–4.2%

**Note.** N = evaluable items per model within each tier (variables × 91 articles): low, 7 × 91 = 637; medium, 8 × 91 = 728; high, 7 × 91 = 637. Total N = 2002 per model. Models ranked by accuracy within each tier. 95% CI = Wilson score interval. Between-model differences: low Cochran’s Q = 14.52 *p* = 0.002; medium Q = 103.73 *p* < 0.0001; high Q = 95.23 *p* < 0.0001.

**Table 3:** **Error taxonomy by model**.

Model	Total N	Correct (n)	Accuracy (%)	Errors (n)	Omissions (n)	Omissions (% of Errors)	Misclassification (n)	Misclassification (% of Errors)
**Gemini 3 Pro Preview**	2002	1129	56.4%	873	92	10.5%	781	89.5%
**Sonar Pro**	2002	1044	52.1%	958	80	8.4%	878	91.6%
**Claude Opus 4.5**	2002	1027	51.3%	975	26	2.7%	949	97.3%
**GPT 5.2**	2002	930	46.5%	1072	101	9.4%	971	90.6%

**Note.** N = 2002 evaluable items per model (91 articles × 22 variables). Errors = total incorrect extractions (N − Correct). Omission = model returned “not reported” when ground truth contained a valid value. Misclassification = incorrect value provided. Percentages for omission and misclassification are reported as proportions of total errors, not total items. Hallucination was not assessed in this study. Models ranked by overall accuracy.

## Data Availability

The data presented in this study are available from the corresponding author upon request.

## Supplementary Materials

The following supporting information can be downloaded at: https://www.mdpi.com/article/10.3390/jcm15114230/s1, Supplementary Table S1: Semantic match analysis for main performance metric; Supplementary Table S2: Mixed-effects logistic regression of extraction accuracy; Supplementary Table S3: Article-level comparison of ground truth and LLM extractions; Supplementary Data S1: Full extraction prompt with field definitions and normalization rules.

## Abbreviations

The following abbreviations are used in this manuscript:

AI	Artificial intelligence
CI	Confidence interval
CLAIM	Checklist of AI in Medical Imaging
GT	Ground truth
JSON	JavaScript Object Notation
LLM	Large language model
SD	Standard deviation
TRIPOD+AI	Transparent reporting of a multivariable prediction model for individual prognosis or diagnosis + artificial intelligence
