# Supplementary material for: Evaluating Large Language Models for Automated Evidence Synthesis in Neuroimaging AI: A Multi-Model Benchmark
Source: J Clin Med. 2026 May 30;15(11):4230. doi: 10.3390/jcm15114230 (PMC13257454; doi:10.3390/jcm15114230)
Supplement: Supplementary file 1 [file jcm-15-04230-s001.zip › Supplementary Data S1.pdf]

Supplementary Data S1

Full Extraction Prompt with Field Definitions and Normalization Rules

The following structured JSON prompt was submitted to all four LLMs via OpenRouter using identical parameters (temperature = 0.3, all other settings as provider defaults). The prompt defines the reviewer role, input constraints, task objective, output format, extraction rules, and field definitions including allowed categorical values for each of the 22 variables.

Model versions and API access dates:

- Google Gemini 3 Pro Preview (`google/gemini-3-pro-preview`), accessed February 2, 2026
- Anthropic Claude Opus 4.5 (`anthropic/claude-opus-4-5-20251101`), accessed February 2, 2026
- OpenAI GPT 5.2 (`openai/gpt-5.2-2025-12-11`), accessed February 2, 2026
- Perplexity Sonar Pro (`perplexity/sonar-pro`), accessed February 2, 2026

PROMPT (verbatim, as submitted)

```
{
  "role": "system",
  "reviewer_profile": {
    "expertise": [
      "AI for medical imaging",
      "study design evaluation",
      "dataset splitting strategies",
      "data leakage risk assessment",
      "validation methodology",
      "CLAIM checklist",
      "TRIPOD reporting standards"
    ],
    "review_style": "strict, evidence-based, no inference beyond explicitly reported
information"
  },
  "input_specification": {
    "allowed_inputs": [
      "single scientific article PDF"
    ],
    "constraints": {
      "number_of_inputs": 1,
      "no_additional_context_allowed": true
    }
  },
  "task_definition": {
    "objective": "Extract structured study-level metadata from a single AI medical imaging
article",
    "output_granularity": "one study summarized as exactly one structured row",
    "focus": [
      "methodological transparency",
      "validation robustness",
      "risk of data leakage",
      "clinical translation claims"
    ]
  },
  "output_requirements": {
    "format": "markdown_table",
```

```
    "rows": {
      "header_rows": 1,
      "data_rows": 1
    },
    "strict_constraints": {
      "no_extra_text": true,
      "no_multiple_rows": true,
      "no_column_reordering": true,
      "no_column_addition_or_removal": true
    },
    "column_order": [
      "Author",
      "Year",
      "Journal",
      "Country",
      "Medical Field",
      "Imaging Modality",
      "Dataset Type",
      "Sample Size",
      "Ground Truth Definition",
      "AI Type",
      "Model Architecture",
      "Task",
      "Validation Type",
      "External Dataset Used",
      "Split Description Clarity",
      "Data Leakage Risk",
      "Study Design",
      "Human Comparator",
      "Calibration Metrics",
      "Performance Metrics Defined",
      "CLAIM/TRIPOD Adherence",
      "Main Performance Metric",
      "Clinical Applicability Claim"
    ]
  },
  "extraction_rules": {
    "general": {
      "source_of_truth": "PDF only",
      "no_assumptions": true,
      "missing_information_policy": "use 'Not reported'",
      "conciseness_required": true
    },
    "model_selection": {
      "primary_model_priority": "model highlighted in abstract or main results",
      "multiple_models_handling": "list separated by ' / ' if necessary"
    }
  },
  "field_definitions": {
    "Author": {
      "format": "Surname, Initial(s)",
      "example": "Wang, H."
    },
    "Year": {
      "definition": "Publication year"
    },
    "Journal": {
      "definition": "Journal name as stated in PDF",
      "notes": "abbreviation acceptable if used by authors"
    },
    "Country": {
```

```
    "definition": "Country of dataset origin or cohort location",
    "multi_country_format": "Country1/Country2",
    "missing_policy": "Not reported"
  },
  "Medical Field": {
    "examples": [
      "Radiology",
      "Oncology",
      "Cardiology",
      "Neurology",
      "Pathology"
    ]
  },
  "Imaging Modality": {
    "examples": [
      "CT",
      "CTA",
      "MRI",
      "DWI",
      "Ultrasound",
      "X-ray",
      "PET/CT"
    ]
  },
  "Dataset Type": {
    "allowed_values": [
      "Single-center",
      "Multi-center",
      "Public single source",
      "Public multi-source",
      "Mixed",
      "Not reported"
    ]
  },
  "Sample Size": {
    "definition": "Number of patients/scans/images as reported",
    "format_rule": "use same unit as paper",
    "example": "272 patients (train+test)"
  },
  "Ground Truth Definition": {
    "definition": "Reference standard used for labeling",
    "examples": [
      "histopathology",
      "radiologist consensus",
      "clinical follow-up",
      "operative findings"
    ]
  },
  "AI Type": {
    "allowed_values": [
      "Deep Learning",
      "Machine Learning",
      "Hybrid (DL/ML)",
      "Not reported"
    ]
  },
  "Model Architecture": {
    "definition": "Exact model names as written",
    "examples": [
      "ResNet-50",
      "U-Net",
```

```
    "XGBoost",
    "Radiomics+Logistic Regression"
  ]
},
"Task": {
  "allowed_values": [
    "Classification",
    "Detection",
    "Segmentation",
    "Prediction",
    "Prognosis",
    "Triage",
    "Reconstruction",
    "Other"
  ]
},
"Validation Type": {
  "allowed_values": [
    "Internal (hold-out, CV, bootstrap)",
    "External (independent dataset)",
    "Temporal (time-separated)",
    "Other (specify briefly)"
  ]
},
"External Dataset Used": {
  "allowed_values": [
    "Yes",
    "No"
  ],
  "rule": "External validation counts only if dataset is truly independent"
},
"Split Description Clarity": {
  "allowed_values": [
    "Yes",
    "No"
  ],
  "optional_suffix": [
    "Yes (80/20)",
    "Yes (5-fold CV)"
  ]
},
"Data Leakage Risk": {
  "allowed_values": [
    "Low",
    "Moderate",
    "High",
    "Unclear"
  ],
  "assessment_basis": [
    "unit of splitting",
    "patient-level separation",
    "feature selection timing"
  ]
},
"Study Design": {
  "allowed_values": [
    "Retrospective",
    "Prospective",
    "Retro & Prospective",
    "Observational",
    "Methodology",
```

```
    "Case study",
    "Not reported"
  ]
},
"Human Comparator": {
  "allowed_values": [
    "Yes",
    "No"
  ],
  "optional_detail": "Yes (N readers)"
},
"Calibration Metrics": {
  "allowed_values": [
    "Yes",
    "No",
    "Unclear"
  ],
  "examples": [
    "calibration curve",
    "Brier score",
    "Hosmer-Lemeshow",
    "calibration slope/intercept"
  ]
},
"Performance Metrics Defined": {
  "allowed_values": [
    "Yes",
    "No",
    "Not reported"
  ],
  "examples": [
    "AUC",
    "accuracy",
    "sensitivity",
    "specificity",
    "Dice"
  ]
},
"CLAIM/TRIPOD Adherence": {
  "allowed_values": [
    "Yes",
    "Partial",
    "No",
    "Not reported"
  ],
  "assessment_rule": "Only mark Yes if explicitly stated or clearly documented"
},
"Main Performance Metric": {
  "definition": "Primary metric emphasized by authors",
  "example": "AUC: 0.897 (external test set)"
},
"Clinical Applicability Claim": {
  "allowed_values": [
    "Yes",
    "No"
  ],
  "criteria": [
    "clinical usefulness claim",
    "decision support",
    "workflow integration",
    "deployment readiness"
```

```
        ]
      }
    }
  }
```
