# Supplementary material for: Evaluating Large Language Models for Automated Evidence Synthesis in Neuroimaging AI: A Multi-Model Benchmark
Source: J Clin Med. 2026 May 30;15(11):4230. doi: 10.3390/jcm15114230 (PMC13257454; doi:10.3390/jcm15114230)
Supplement: Supplementary file 1 [file jcm-15-04230-s001.zip › Supplementary Table S1.pdf]

Supplementary Table S1. Semantic Match Analysis for Main Performance Metric.

Model	Total Items (N)	Exact (n)	Exact %	Semantic+ (n)	Semantic+ %	95% CI (Wilson)
Claude Opus 4.5	91	0	0.0%	89	97.8%	92.3–99.4%
GPT 5.2	91	0	0.0%	86	94.5%	87.8–97.6%
Gemini 3 Pro Preview	91	0	0.0%	83	91.2%	83.6–95.5%
Sonar Pro	91	0	0.0%	81	89.0%	80.9–93.9%

Note. N = 91 articles per model. Exact = character-for-character match with ground truth. Semantic+ = exact match plus semantically equivalent responses. 95% CI = Wilson score interval. Models ranked by Semantic+ rate.
