# Supplementary material for: Evaluating Large Language Models for Automated Evidence Synthesis in Neuroimaging AI: A Multi-Model Benchmark
Source: J Clin Med. 2026 May 30;15(11):4230. doi: 10.3390/jcm15114230 (PMC13257454; doi:10.3390/jcm15114230)
Supplement: Supplementary file 1 [file jcm-15-04230-s001.zip › Supplementary Table S2.pdf]

Supplementary Table S2. Mixed-Effects Logistic Regression of Extraction Accuracy.

Parameter	Estimate (log-odds)	Odds Ratio	95% CI	p-value
Fixed Effects				
Model effects (reference: GPT 5.2)		***1.00***	—	—
Claude Opus 4.5	0.372	1.45	1.29–1.64	<0.0001
Sonar Pro	0.438	1.55	1.37–1.75	<0.0001
Gemini 3 Pro Preview	0.774	2.17	1.92–2.45	<0.0001
Complexity (reference: Low)		***1.00***	—	—
Medium	−2.201	0.11	0.10–0.12	<0.0001
High	−5.273	0.005	0.004–0.006	<0.0001
Intercept (Low complexity, GPT 5.2)	2.064	7.88	7.42–8.38	<0.0001
Random Effects (Variance Components)				
Article (random intercept)	0.160	—	—	—
Variable (random intercept)	1.201	—	—	—

Note. Model: Bayesian variational-Bayes logistic mixed-effects regression of correct extraction (binary outcome) on AI model and variable complexity tier as fixed effects, with crossed random intercepts for article (n = 91) and variable (n = 22). N = 8,008 observations (4 models × 2,002 items). Odds ratios are reported relative to the reference category (GPT 5.2 for model contrasts; Low complexity for complexity contrasts). 95% confidence intervals derived from posterior standard deviations. The dominance of variable-level variance (1.20 log-odds²) over article-level variance (0.16) indicates that extraction difficulty depends more strongly on the variable being extracted than on the source article, consistent with the variable-complexity stratification used throughout the manuscript.
